# Supplementary material for: Diploid genome assembly of the Malbec grapevine cultivar enables haplotype-aware analysis of transcriptomic differences underlying clonal phenotypic variation
Source: Hortic Res. 2024 Mar 14;11(5):uhae080. doi: 10.1093/hr/uhae080 (PMC11101320; doi:10.1093/hr/uhae080)

**Supplementary Figure 2**. Contiguity (N50) of the obtained contigs and scaffolds, compared to the phased blocks for **(A)** Malbec-Mag and **(B)** Malbec-Pru haplophases. Phased blocks contiguity was estimated after mapping the parental reads k-mers (hap-mers) to the respective assembly.


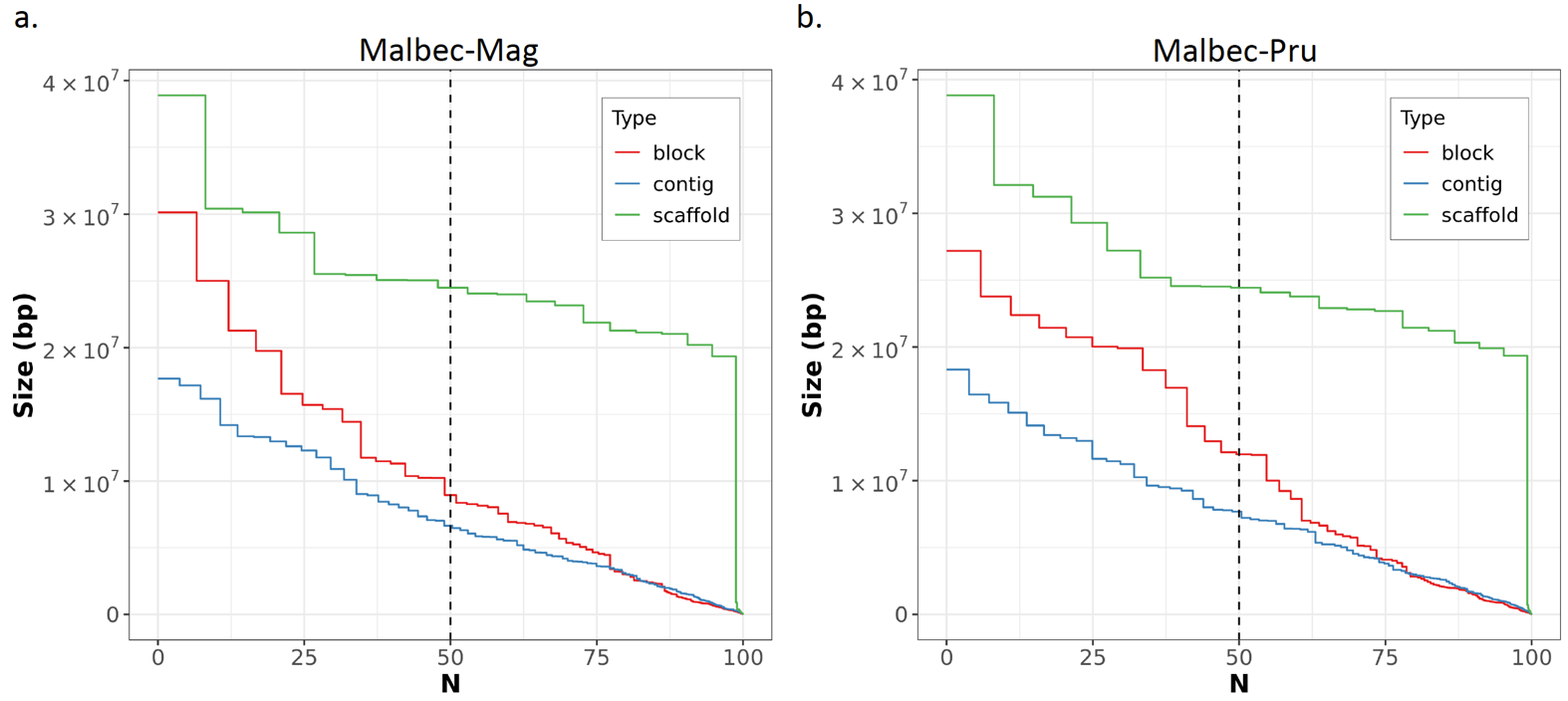

Supplement: Web_Material_uhae080 [file web_material_uhae080.zip › Figure_S2.docx]
