# Supplementary material for: Diploid genome assembly of the Malbec grapevine cultivar enables haplotype-aware analysis of transcriptomic differences underlying clonal phenotypic variation
Source: Hortic Res. 2024 Mar 14;11(5):uhae080. doi: 10.1093/hr/uhae080 (PMC11101320; doi:10.1093/hr/uhae080)
Supplement: Web_Material_uhae080 [file web_material_uhae080.zip › Figure_S6.docx]

**Supplementary Figure 6.** Pairwise comparisons based on global transcriptomic sample-to-sample distance. Pairwise comparisons were performed with Malbec-Mag (A to C) and Malbec-Pru (D to F). The obtained results were identical with either haplophase, in all cases Malbec clone accession 595 differentiated from the other accessions.
